# The *Shewanella algae* strain YM8 produces volatiles with strong inhibition activity against *Aspergillus* pathogens and aflatoxins

**DOI:** 10.3389/fmicb.2015.01091

**Published:** 2015-10-06

**Authors:** An-Dong Gong, He-Ping Li, Lu Shen, Jing-Bo Zhang, Ai-Bo Wu, Wei-Jie He, Qing-Song Yuan, Jing-De He, Yu-Cai Liao

**Affiliations:** ^1^Molecular Biotechnology Laboratory of Triticeae Crops, Huazhong Agricultural UniversityWuhan, China; ^2^College of Plant Science and Technology, Huazhong Agricultural UniversityWuhan, China; ^3^College of Life Science and Technology, Huazhong Agricultural UniversityWuhan, China; ^4^Key Laboratory of Food Safety Research Institute for Nutritional Sciences, Shanghai Institutes for Biological Sciences, Chinese Academy of SciencesShanghai, China; ^5^National Center of Plant Gene Research (Wuhan), Huazhong Agricultural UniversityWuhan, China

**Keywords:** aflatoxins, antifungal, *Aspergillus* species, marine bacterium, *Shewanella algae*, VOCs, volatiles

## Abstract

Aflatoxigenic *Aspergillus* fungi and associated aflatoxins are ubiquitous in the production and storage of food/feed commodities. Controlling these microbes is a challenge. In this study, the *Shewanella algae* strain YM8 was found to produce volatiles that have strong antifungal activity against *Aspergillus* pathogens. Gas chromatography-mass spectrometry profiling revealed 15 volatile organic compounds (VOCs) emitted from YM8, of which dimethyl trisulfide was the most abundant. We obtained authentic reference standards for six of the VOCs; these all significantly reduced mycelial growth and conidial germination in *Aspergillus*; dimethyl trisulfide and 2,4-bis(1,1-dimethylethyl)-phenol showed the strongest inhibitory activity. YM8 completely inhibited *Aspergillus* growth and aflatoxin biosynthesis in maize and peanut samples stored at different water activity levels, and scanning electron microscopy revealed severely damaged conidia and a complete lack of mycelium development and conidiogenesis. YM8 also completely inhibited the growth of eight other agronomically important species of phytopathogenic fungi: *A. parasiticus, A. niger, Alternaria alternate, Botrytis cinerea, Fusarium graminearum, Fusarium oxysporum, Monilinia fructicola*, and *Sclerotinia sclerotiorum*. This study demonstrates the susceptibility of *Aspergillus* and other fungi to VOCs from marine bacteria and indicates a new strategy for effectively controlling these pathogens and the associated mycotoxin production during storage and possibly in the field.

## Introduction

Aflatoxigenic *Aspergillus* fungi are ubiquitous pathogens that infect many crops and also contaminate food/feed commodities during storage (Kamika and Takoy, 2011; Asters et al., 2014). *A. flavus* and *A. parasiticus* are the predominant species; the former species is second only to *A. fumigatus* as the cause of human invasive aspergillosis (Amaike and Keller, 2011) and is the main *Aspergillus* species that infects insects (Hedayati et al., 2007). These fungi can produce various types of aflatoxins that have been demonstrated to be carcinogenic in many animal species (Amaike and Keller, 2011; Waliyar et al., 2015). Consumption of high doses of aflatoxins is fatal (acute aflatoxicoses), while chronic exposure to small quantities may lead to liver cancer or liver cirrhosis (Waliyar et al., 2015). The International Agency for Research on Cancer has classified naturally occurring aflatoxins as Group 1 human carcinogens (Williams et al., 2004). Currently, more than 5 billion people worldwide suffer from uncontrolled exposure to aflatoxins (Williams et al., 2004). Outbreaks of acute aflatoxicosis in developing countries are increasing in frequency and cause unacceptably high numbers of deaths (Amaike and Keller, 2011; Waliyar et al., 2015). The most effective strategy to reduce and/or eliminate aflatoxins is to prevent aflatoxigenic *Aspergillus* fungi from infecting crops in the field and to prevent the infection of food/feed products during storage. Thus, characterization of a novel microorganism with inhibition activity against *Aspergillus* fungi and aflatoxin biosynthesis is vitally important for reducing aflatoxin content in food/feed chains.

To date, fungicides have been the primary means of control used for aflatoxigenic *Aspergillus* pathogens, as crop cultivars resistant to these pathogens are not adequately available (Torres et al., 2014). The direct application of fungicides to harvested crops or to food/feed products in storage can be deleterious to human and livestock health (Torres et al., 2014), can lead to serious environmental hazards, and may even lead to enhanced aflatoxin production (Santos et al., 2011). Thus, alternative strategies such as bio-control agents have been sought and some have been developed. For instance, two non-toxigenic *Aspergillus* strains have been registered in the United States to control aflatoxins (Dorner and Cole, 2002; Dorner, 2004). *Bacillus megaterium* (Kong et al., 2010) and *B. pumilus* (Cho et al., 2009), have been shown to control the growth of *Aspergillus* fungi. In addition, volatile compounds from mushroom (Petrovic et al., 2013) and plants such as *Peumus boldus, Lippie turbinate* (Passone and Etcheverry, 2014), *Cuminum cyminum* (Kedia et al., 2014), and soybean (Cleveland et al., 2009) have been reported to be useful in the control of *Aspergillus*. Research conducted in the last decade has shown that certain bacterial volatiles have antifungal activity against a range of phytopathogenic organisms (Kai et al., 2009; Effmert et al., 2012). However, the effectiveness of bacterial volatile compounds in reducing *Aspergillus* infection and aflatoxin contamination remain unknown.

Species of the genus *Shewanella* are aquatic microorganisms widely distributed in marine and fresh water environments (Hau and Gralnick, 2007). These gram negative bacteria can respire with a diverse array of electron acceptors, and have adapted to life in extreme and varied environments. They have shown great potential for remediation of various environmental pollutants such as radionuclides, toxic elemental waste, halogenated organic compounds, and cyclic nitramines. They have also shown potential for use in biofuel cell applications (Hau and Gralnick, 2007). No previous study has reported that *Shewanella* bacteria or their volatiles have antifungal activity against pathogens.

In this study, we identified the marine *Shewanella algae* strain YM8 that produces volatiles with strong inhibition activity against *Aspergillus* pathogens. Our objectives were to determine the chemical structures and modes of inhibition of these volatile organic compounds (VOCs) and to test the *in vivo* efficacy of these compounds against *Aspergillus* growth and aflatoxin biosynthesis in agricultural samples. Strain YM8 produced 15 VOCs including the well-known antimicrobial compounds dimethyl trisulfide and 2,4-bis(1,1-dimethylethyl)-phenol, the registered fungicide butylated hydroxytoluene, and several novel bioactive compounds. Strain YM8 not only completely inhibited *Aspergillus* mycelial growth and conidial germination but also completely inhibited the growth of eight other species of phytopathogenic fungi. This newly identified marine bacterium may find wide application for the control of phytopathogenic fungi in the field and during storage, and should prove extremely useful in the dissection of the molecular mechanism underlying the inhibition of a wide spectrum of fungal species.

## Materials and methods

### Microorganisms and crop kernels

Strain YM8 isolated from sea sediment in east China coast was cultured in nutrient broth (NB) medium (Fang et al., 2014) for maintenance, and spread on nutrient agar (NA) plates (Gong et al., 2015) for producing VOCs. Bacterial cultures were maintained as 25% glycerol stocks at −80°C for long-term storage.

The nine agronomically-important phytopathogenic species of fungi in this study were from our laboratory, and included *Aspergillus flavus* (*AF*), *Aspergillus parasiticus, Aspergillus niger, Alternaria alternate, Botrytis cinerea, Fusarium oxysporum, Fusarium graminearum, Monilinia fructicola*, and *Sclerotinia sclerotiorum*. Fungal strains were inoculated on potato dextrose agar (PDA) plates (Gong et al., 2015) for growth inhibition tests. *AF* is an aflatoxigenic producer that was originally isolated from diseased peanuts in Wuhan, China (Xue et al., 2013). Fresh *AF* conidia on the surface of the PDA plates were washed off with sterilized water and filtrated through two layers of gauze. Conidia were stored in 10% (v/v) glycerol at –20°C and used within 2 weeks of sampling.

Mature, healthy, regular-sized grains of maize (Zhengdan 958) and peanuts (Silihong) were inoculated with *AF*, and challenged with YM8 for bio-control assays.

### DNA extraction and phylogenetic alignment

Strain YM8 was cultured on NB medium at 28°C with shaking at 200 rpm for 48 h. Genomic DNA was extracted and 16S rDNA sequences were amplified by PCR (Gong et al., 2015). PCR products were sequenced and submitted to the NCBI database. A phylogenetic tree of YM8 and homologous strains was constructed with a neighbor-joining method (Ding et al., 2015).

### Inhibition of strain YM8 against mycelial growth and conidial germination of *AF*

The effect of strain YM8 against the mycelial growth and conidial germination of *AF* was conducted in two inverse face-to-face petri dishes (Fernando et al., 2005) (90 mm in diameter) with a 0.2 L airspace. Aliquots of 100 μl YM8 suspension (10^8^ cfu/ml) were smeared over NA plates. *AF* mycelium was inoculated on PDA plates. The two dishes were placed face-to-face, sealed with scotch tape, and incubated at 28°C for 5 days. *AF* mycelium co-cultured on NA plates smeared with NB medium was used as a control. The inhibition rate was calculated using the following formula: Inhibition rate (%) = [(the diameter of control – the diameter of antagonist treatment)/the diameter of control] × 100.

The effect of strain YM8 against conidial germination was conducted as described above in sealed petri dishes where 100 μl of conidia at 5 × 10^5^ cfu/ml in water were spread on PDA plate. Two face-to face dishes were cultured at 28°C for 2 days.

### Effect of charcoal on the inhibitory activity of strain YM8

Active charcoal, a reagent that absorbs VOCs, was used in the YM8 and *AF* assays (Fernando et al., 2005). One dish (90 mm in diameter) was equally divided into two parts, only one part contained NA medium. Charcoal (5 g) and strain YM8 inoculum (in the half plate with NA medium) were placed in the divided dish. *AF* inoculated on the center of PDA plates (90 mm in diameter) was set above the divided plate. The two plates were sealed and cultured at 28°C for 5 days. There were four treatments including: (i) *AF* alone; (ii) *AF* + charcoal; (iii) *AF* + strain YM8; and (iv) *AF* + strain YM8 + charcoal. The inhibition rate was calculated using the formula mentioned above.

### Identification of volatile compounds

Strain YM8 (50 μl at 10^8^ cfu/ml) was spread on NA medium surface (40 ml) in a 100 ml flask. The flask was sealed from air exchange with two layers of plastic membrane. Flasks containing uninoculated NA medium were used as controls. The flasks were cultured at 28°C and in the dark for 48 h. Before sampling, the flasks were transferred to a bath of 40°C water and allowed to equilibrate for 30 min. A 50/30 μm solid-phase micro-extraction (SPME) fiber containing divinylbenzene/carboxen/polydimethlsiloxane was used to extract the volatiles, and the extracted volatiles were analyzed based on previously described methods with a GC-MS (5975B-7890N, Agilent Technologies Inc.) instrument equipped with an Agilent DB-5MS fused-C18 capillary column (Huang et al., 2011; Yuan et al., 2012; Cernava et al., 2015). The identified compounds from the YM8 samples that were not present in the control samples were considered to be the final analyte compounds.

### Inhibitory effects of VOCs against the mycelial growth and conidial germination of *AF*

Authentic reference standard compounds were purchased for six of the final analyte compounds identified by GC-MS (Table S1) and individually tested against *AF*. *AF* mycelial plugs were inoculated at the center of PDA plates, and the another dish containing paper disk that had been inoculated with 40 μl of diluted reference standard volatiles for final concentrations (compound weight to airspace volume) of 5, 10, 100, and 200 μg/L. Disks inoculated with 40 μl of ethanol were used as controls. The *AF* and the disk-containing dishes were placed face-to-face, sealed, and cultured at 28°C in the dark (Li et al., 2010); growth was measured at 5 days post inoculation (dpi) and the inhibition rate was calculated using the formula mentioned above.

For the conidial germination test, 100 μl *AF* conidia suspension of 5 × 10^5^ cfu/ml was spread on PDA plates. Dishes containing the disks with each of the standard volatile compounds were co-cultured with conidium plates. Each pair of dishes was conducted as described for mycelial assays and placed at 28°C for 24 h. Conidia were then assayed under a microscope and the conidial germination rate was calculated with this formula: Inhibition rate (%) = [(the germinated conidia of control – the germinated conidia of antagonist treatment)/the germinated conidia of control] × 100.

### Microbes management of *AF* on peanuts and maize using strain YM8

Bio-control activity assays of YM8 against *AF* were conducted at three different water activities (a_w_) in a sealed desiccator (2.5 L airspace). Fifty gram of peanut or maize grains in 250 ml flasks were autoclaved at 121°C for 20 min. After cooling, each flask was inoculated with 1 ml of a 5 × 10^5^ cfu/ml suspension of *AF* conidia. Sterilized water was added to each flask to adjust a_w_ to 0.740, 0.859, and 0.923 for corn, and to 0.785, 0.866, and 0.934 for peanuts. The a_w_ levels of maize and peanut were determined at 28°C (±0.2°C) using an electronic dewpoint water activity meter, Aqualab Series 3 model TE (Decagon Devices, Pullman, Washington, USA). The samples at each a_w_ level were equally divided into two parts; half of the sample was challenged with strain YM8 (the bacteria were grown on NA plate with 15 cm in diameter) and cultured in a desiccator and the other half was treated with NA medium as a control and cultured in a separate desiccator. The samples in each treatment were collected on day 7 of the assay and dried at 60°C for 5 days. The dried samples were milled and used for aflatoxin analyses.

The effect of strain YM8 volatiles on the germination of maize and peanut seeds was determined. After co-cultured with YM8 for 7 days, the seeds without autoclaving were surface-sterilized with HgCl_2_ (0.1%, v/v) for 5 min, and washed 3 times with sterilized water; the seeds were placed on wet filters in petri dishes and cultured at 28°C for 4 days, at which time the seed germination rate was calculated.

### Analysis of aflatoxins in infected peanuts and maize

Aflatoxins in milled maize and peanuts were extracted with previously described method (Warth et al., 2012). One gram of each sample was placed into a 5 ml polypropylene tube and extracted with 5 ml acetonitrile/water (84/16, v/v). The samples were mixed for 1 min on vortex, and then sonicated for 60 min. After centrifugation, 1 ml of supernatant was transferred into new tubes and 1 ml of hexane was added. Following a 10 min extraction, 500 μl of the upper (organic) layer was sampled and used for LC-ESI-MS aflatoxin analysis with a Thermo Surveyor plus HPLC system coupled to a TSQ Quantum Ultra mass spectrometer (Thermo Scientific, CA, USA) (Warth et al., 2012). The mobile phase consisted of eluent A (MeOH) and eluent B (water containing 5 mM ammonium acetate and 0.05% formic acid); the flow rate for the analysis was 0.3 mL/min. The linear gradient program was as follows: 0–1 min, 20% A; 1–4 min, 20–100% A; 4–5 min, 100% A; 5–5.5 min, 100%–20% A; 5.5–7 min, 20% A, giving a total analysis time of 7 min. Authentic reference standard aflatoxin compounds (AFB1, AFB2, AFG1, and AFG2) were purchased from Sigma (Sigma-Aldrich, St. Louis, MO, USA) and used as standards for identification and quantification.

### Ultra-structure of *AF* on infected peanuts

Conidia and infection structures on infected peanuts at a_w_ 0.932 were examined with SEM. *AF*-infected peanuts treated with YM8 and controls were fixed with 1% osmic acid for 1 h. A small piece of the peanut coat about 5 × 5 mm was peeled off under a stereo microscopy (Olympus SZX16, Tokyo, Japan) (Jabeen et al., 2012) and affixed to SEM stubs. Samples were then coated with gold and examined with a JSM-6390 SEM (Hitachi, Tokyo, Japan) (Boukaew and Prasertsan, 2014).

### Temperature stability assays of YM8 antifungal activity and antifungal activity of YM8 against selected phytopathogenic fungi

The temperature range of antifungal activity was tested in face-to-face petri dishes as described above at 10, 20, 30, and 40°C. The diameter of *AF* mycelial growth was measured at 5 dpi. Antifungal activity of YM8 against eight selected phytopathogenic fungal species was assayed as described for the *AF* mycelia.

### Data analysis

Data were evaluated with analysis of variance (ANOVA) using SAS 9.2 for windows (SAS Institute, Cary, NC, USA). Experiments were arranged in a completely randomized design with three replications. Mean comparisons were performed with Duncan's multiple range tests at the *P* < 0.01 or 0.001 levels.

## Results

### Inhibition of *Aspergillus flavus* by volatiles from *Shewanella algae* strain YM8

**Figure 1:**
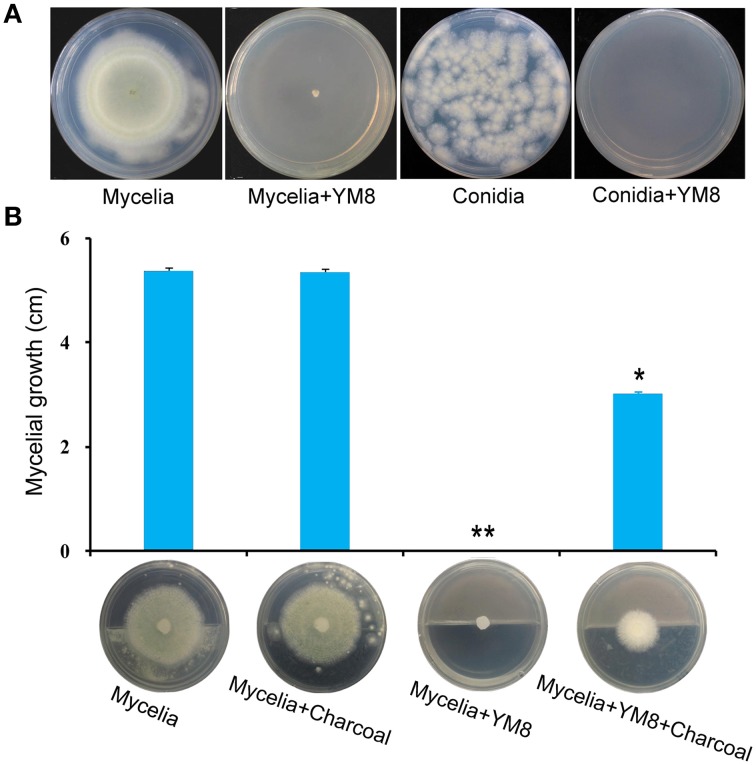
**Inhibitory effect of volatiles from bacterial strain YM8 on the mycelial growth and conidial germination of ***Aspergillus flavus***. (A)** Mycelia and conidia of *A. flavus* growing on potato dextrose agar (PDA) in the presence or absence of YM8. **(B)** Mycelial growth in the presence of YM8 alone or YM8 plus charcoal. Data represent the means ± SE from three measurements. ^**^P < 0.001, ^*^P < 0.01.

Initial screening of antagonistic bacteria revealed that strain YM8 isolated from marine sediment showed antifungal activity against *A. flavus*. Further assays in sealed petri dishes with 0.2 L of airspace suggested that this antifungal activity may be due to volatiles emitted from the YM8 strain, as there was no physical contact between *AF* and YM8. As shown in Figure 1, untreated *AF* mycelia grew to a diameter of 5.4 cm 5 days post inoculation (dpi) and had conidia that germinated within 48 h on PDA media, whereas mycelial growth and germination of conidia were completely inhibited in the presence of strain YM8. To prove that volatiles were directly responsible for the observed antifungal activity, charcoal was added to the strain YM8 and *AF* plates to adsorb volatiles. The presence of charcoal significantly reduced (60% reduction) the antifungal activity of strain YM8, indicating that the antifungal activity against *AF* was caused by volatiles produced by strain YM8.

**Figure 2:**
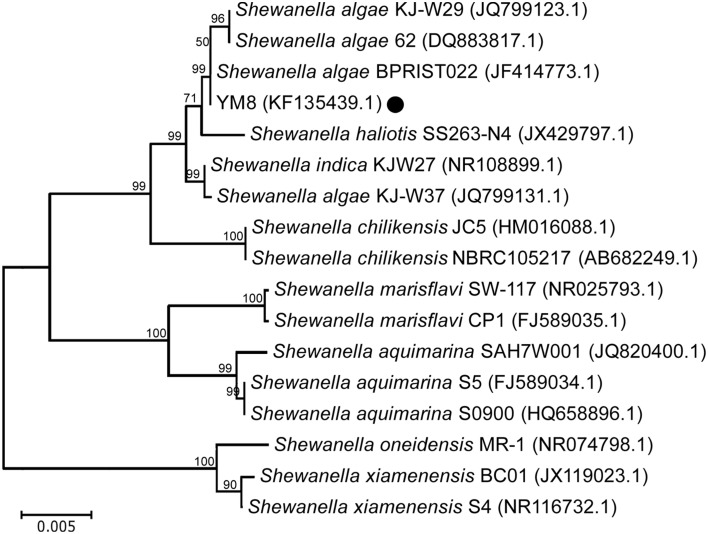
**Neighbor-joining phylogenetic tree based on 16S rDNA sequences of the YM8 strain and other ***Shewanella*** spp. retrieved from the NCBI database**. The scale bar represents the number of substitutions per base position.

BLAST analysis of the strain YM8 16S rDNA sequence (GenBank ID No. KF135439) against other sequences from the Nucleotide Sequence Database of NCBI (National Center for Biotechnology Information) revealed that strain YM8 is a strain of the bacterial species *S. algae* (Figure 2). Phylogenetic analyses revealed that strainYM8 is in the same sub-cluster with the *S. algae* strain BPRIST022 and close to two *S. algae* strains, and that these were all similar to other species of the *Shewanella* genus. The results clearly indicate that the *S. algae* marine bacterium strain YM8 can produce volatiles with strong inhibitory activity against *AF* mycelial growth and conidial germination.

### Chemical identification of the volatiles produced by *S. algae* strain YM8

**Figure 3:**
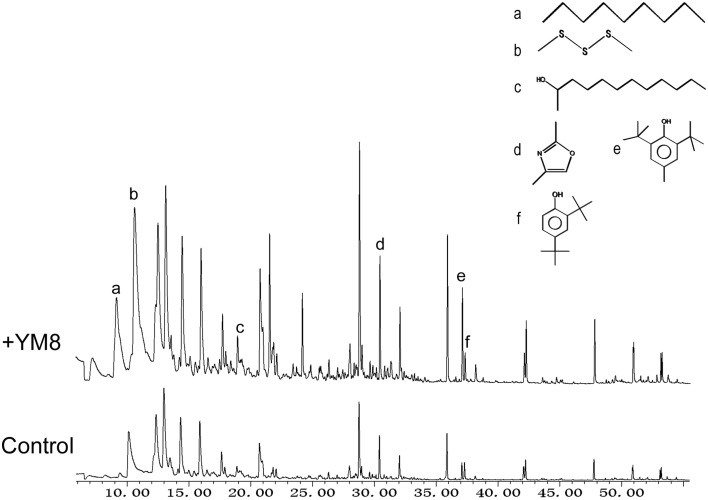
**GC-MS spectra of volatiles emitted from strain YM8**. Bacterial strain YM8 grown on NA plates for 48 h and control NA medium. Detected volatile organic compounds (VOCs) are listed in Table 1. Chemical structures of six selected VOCs: a, nonane; b, dimethyl trisulfide; c, 2-dodecanol; d, 2,4-dimethyl-oxazole; e, butylated hydroxytoluene; f, 2,4-bis(1,1-dimethylethyl)-phenol.

We used GC-MS to profile these antifungal volatile compounds and to attempt structural elucidation. Fifteen VOCs produced by strain YM8 were detected and identified (Figure 3 and Table 1). These VOCs were from several chemical classes, including aromatics, alkanes, sulfides, enols, oxazole, anthracenes, esters, and phenols. The most abundant compound in the volatile profile was identified as dimethyl trisulfide (DMTS). It had a identity (Id) of 98% with entries in the NIST 08 MS spectral database, a molecular mass of 125.963 D, and a retention time of 10.631 min. The DMTS peak area accounted for 14.195% of the total peak area. Four other compounds were moderately abundant: butylated hydroxytoluene (1.533%; SI 97%), 2,4-dimethyl-oxazole (2.307%; SI 80%), 2,4,4,6,6,8,8-heptamethyl-2-nonene (4.976%; SI 56%), and 2,4,4-trimethyl-1-pentanol (1.363%; SI 35%). The remaining 10 volatile compounds identified each accounted for less than 1% of the total peak area. Of the 15 VOCs, six compounds displayed spectral SI greater than70% to entries in the NIST 08 MS database and had peak areas greater than 0.5% of the total peak area in the GC-MS profiling analysis. These six compounds were purchased and used for subsequent antifungal assays (Table S1). The results of the GC-MS profiling analysis indicate that the VOCs emitted from strain YM8 were structurally diverse.

**Table 1:** **GC-MS analysis of volatile organic compounds emitted by strain YM8**.

**No**	**Compounds^a^**	**Area^b^ (%)**	**Id^c^**	**RT^d^ (min)**	**Dalton^e^**
1	dimethyl trisulfide^*^	14.195	98	10.631	125.963
2	2,4,4,6,6,8,8-heptamethyl-2-nonene	4.976	56	30.801	224.25
3	2,4-dimethyl-oxazole^*^	2.307	80	30.424	97.053
4	butylated hydroxytoluene^*^	1.533	97	37.078	220.183
5	2,4,4-trimethyl-1-pentanol	1.363	35	32.043	130.136
6	2,2,4,4,5,5,7,7-octamethyloctane	0.738	35	22.841	226.266
7	methoxy-phenyl-oxime	0.721	91	7.17	151.063
8	2-dodecanol^*^	0.560	81	19.348	186.198
9	nonane^*^	0.536	71	9.899	128.157
10	2,4-bis(1,1-dimethylethyl)-phenol^*^	0.504	96	37.302	206.167
11	2-anthracenamine	0.499	38	10.357	193.089
12	oxalic acid, allyltetradecylester	0.360	47	31.299	326.246
13	fluoren-9-ol, 3,6-dimethoxy-9-(2-phenylethynyl)-	0.315	45	25.526	342.126
14	sulfurous acid, decyl 2-propyl ester	0.244	27	23.689	264.176
15	3-hexen-2-one, 5-methyl-	0.209	43	30.138	112.089

a Fifteen compounds detected in volatiles emitted from strain YM8 growing on NA plate for 48 h. Stars “^*^” represent the authentic reference standard compounds purchased from companies.

b *Relative area of identified compound as a percentage of the total area of the chromatographic peaks*.

c *Identity of analyte compound spectra with entries in the NIST 08 MS spectral database*.

d *Retention time in the GC-MS analysis*.

e *Molecular weight of identified compound*.

### Inhibitory activity of individual volatiles against *AF* mycelial growth

**Figure 4:**
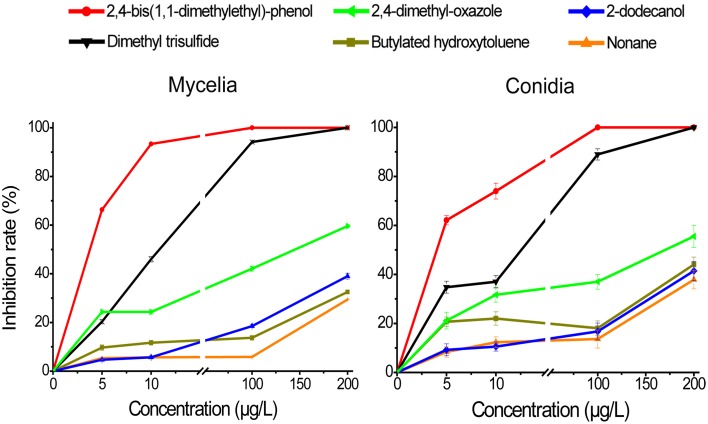
**Inhibition effect of six selected VOC compounds on the mycelial growth and conidial germination of ***Aspergillus flavus*****. Individual compounds with different concentrations were assayed for their inhibition activity against mycelia and conidia of *A. flavus* grown on potato dextrose agar for 5 days (mycelia) and 1 days (conidia). Data represent the means ± SE from three measurements.

To evaluate the antifungal activity of the individual VOCs produced by strain YM8, the six selected compounds were assayed at different concentrations for their inhibitory activity against *AF* mycelial growth 7 dpi on PDA medium (Figure 4). All six compounds could inhibit *AF* mycelial growth at a 5 μg/L concentration (compound weight to airspace volume). Three compounds in particular, DMTS, 2,4-dimethyl-oxazole, and 2,4-bis (1,1-dimethylethyl)-phenol, showed very strong inhibitory activity. In general, inhibition activity increased with increasing concentrations. For both DMTS and 2,4-bis (1,1-dimethylethyl)-phenol, complete inhibition of mycelial growth was observed at 100 μg/L. The remaining three compounds significantly inhibited mycelial growth (32–41%) at 100 μg/L and higher concentrations. These results confirm that some of the VOCs produced by strain YM8 have antifungal activity against *AF* pathogens. Further, the most abundant compound from strain YM8, DMTS, had some of the strongest antifungal properties.

### Inhibitory activity of individual volatiles against the germination of *AF* conidia

Evaluation of the inhibitory effects of the six selected VOC compounds on the germination of conidia revealed a similar pattern as that observed in the mycelial growth assays. Of the six compounds, DMTS and 2,4-bis (1,1-dimethylethyl)-phenol showed the strongest inhibitory activities, with 90% (DMTS) and 100% [2,4-bis (1,1-dimethylethyl)-phenol] inhibition of conidial germination at 100 μg/L (Figure 4). At 200 μg/L, these two compounds completely inhibited the germination of conidia. The remaining four compounds showed differing inhibition rates ranging from 38 to 56%. Thus, all six compounds assayed were able to inhibit both mycelial growth and conidial germination of *AF*.

### Use of strain YM8 for the bio-control of *AF* growth and aflatoxin production in maize and peanut

**Figure 5:**
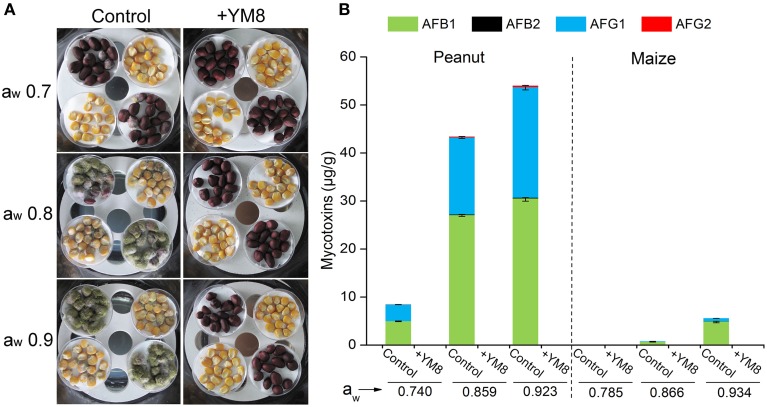
**Efficacy of bacterial strain YM8 for bio-control against ***Aspergillus flavus*** and aflatoxins in peanuts and maize. (A)** Phenotypes of peanuts and maize infected with *Aspergillus flavus* in the presence or absence (control) of strain YM8 at 28°C for 7 days. **(B)** Aflatoxin content in peanuts and maize samples from **(A)**. Data represent the means ± SE from three measurements.

To assess whether strain YM8 could inhibit *AF* growth and mycotoxin biosynthesis in real agricultural samples, strain YM8 was placed together with maize and peanut in enclosed desiccators at three different water activity (a_w_) levels and infected with conidia of an aflatoxigenic *AF* fungus. The a_w_ levels of the environment of stored samples have critically important impacts on *AF* growth and aflatoxin production^14^. The disease incidences and mycotoxin levels of the samples were determined at 7 dpi. As shown in Figure 5, strain YM8 showed very strong inhibitory effects on both fungal growth and aflatoxin production. In peanuts, the control samples had disease incidences of 25, 97, and 100% under conditions with a_w_ of 0.740, 0.859, and 0.923, respectively. The untreated maize controls had diseases incidences of 3, 98, and 100% under conditions with a_w_ of 0.785, 0.866, and 0.934, respectively. In stark contrast, no visible disease symptoms were seen for any of the maize or peanut samples treated with strain YM8 at any of the three a_w_ levels, indicating that the presence of strain YM8 caused complete inhibition of *AF* growth.

To further reveal whether VOCs inhibit the germination of peanut and maize, the purchased peanut and maize seeds directly treated with YM8 were determined for their germination rates. The results showed no significant differences of germination rates between the controls and YM8-treated peanuts or maize, indicating that YM8 had no inhibitory effect on the germination of both peanuts and maize.

Aflatoxin levels in the YM8-treated and control samples were determined by chemical analyses. In the control peanut samples, four aflatoxin compounds (AFB1, AFB2, AFG1, and AFG2) were detected. The summed concentrations of these four compounds at the three different a_w_ levels were 8.5, 43.4, and 54 μg/g, respectively (Figure 5). In contrast, no aflatoxins were detected in any of the peanut samples treated with strain YM8. A similar pattern of results was observed for the maize samples. In the control maize samples, three aflatoxin compounds (AFB1, AFB2, and AFG1) were detected and the summed aflatoxin concentrations of these compounds at the three a_w_ levels were 0.06, 0.8–5.6 μg/g, respectively. No aflatoxins were detected in any of the maize samples treated with strain YM8. These results indicated that the VOCs released from strain YM8 could completely inhibit *AF* growth and aflatoxin production both in different agricultural samples and at different water activity level conditions.

### Scanning electron microscopy images of *AF* treated with YM8 VOCs

**Figure 6:**
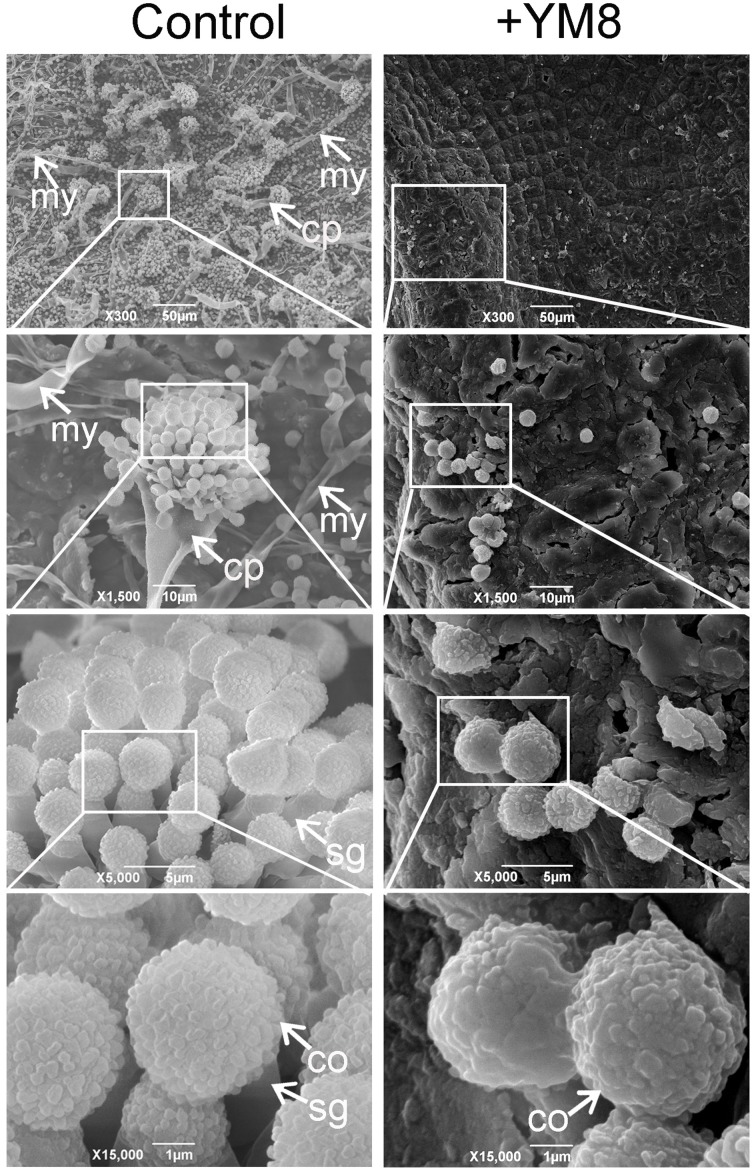
**Scanning electron micoscopy of ***Aspergillus flavus*** infecting peanuts**. Peanuts with water activity (a_w_) of 0.934 were inoculated with *A. flavus* conidia and incubated in the presence or absence (control) of strain YM8 for 7 days. co, conidia; cp, conidiophore; my, mycelia; sg, sterigma.

To further investigate the ultra-structures of *AF* growing on agricultural samples after treatment with YM8, scanning electron microscopy (SEM) was used to examine *AF* structures on peanuts with a_w_ of 0.923 at 7 dpi (Figure 5A). As shown in Figure 6, control peanuts inoculated with conidia were covered with large amounts of *AF* mycelia and had many typical conidiophores with many circular conidia. In samples inoculated with *AF* conidia and treated with YM8, however, there were very few conidia and there were no mycelia or conidiophores; the few conidia that were observed had severely deformed and irregular shapes with curves and holes on the surface. These results indicate that strain YM8 causes severe structural damage to conidia and inhibits the formation of mycelia and conidiophores.

### Temperature stability of inhibitory activity of strain YM8 against *AF*

**Figure 7:**
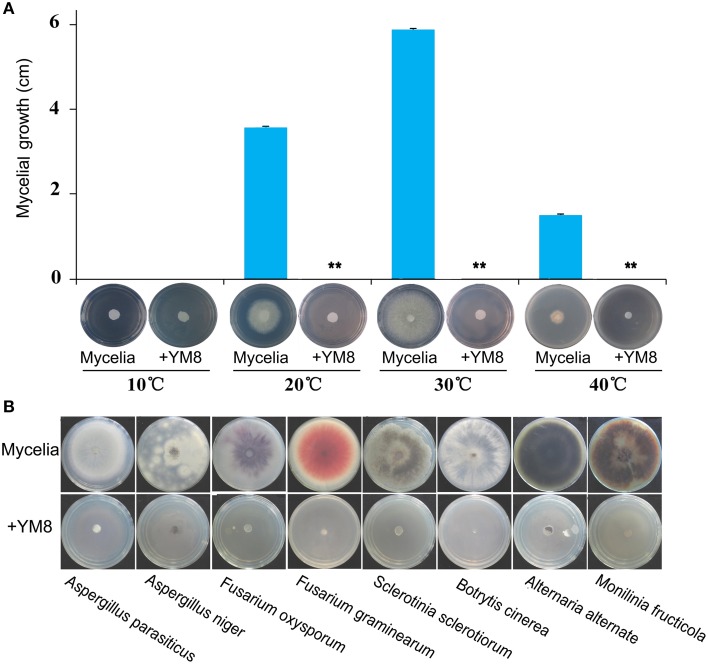
**Temperature stability of strain YM8 antifungal activity and antifungal activity of strain YM8 against selected phytopathogenic fungi. (A)** Range of temperatures for the inhibition of mycelia of *Aspergillus flavus*. **(B)** Phytopathogenic fungal species inhibited by strain YM8. ^**^P < 0.001.

It is important to know whether strain YM8 is able to inhibit *AF* growth and conidial germination under a range of different environmental conditions. We thus tested the inhibitory activity of strain YM8 against *AF* at 10, 20, 30, and 40°C in sealed petri dishes (Figure 7A). Untreated control *AF* grew significantly faster at 30°C (5.88 cm diameter) than at 20°C (3.56 cm) or 40°C (1.50 cm); *AF* did not grow at 10°C. In the presence of strain YM8, there was no *AF* growth in any of the temperature treatments, indicating complete inhibition of growth. These results indicate that strain YM8 can produce VOCs at culture temperatures ranging from 20 to 40°C and that these VOCs are active and efficiently inhibit *AF* growth in this range of temperatures.

### Inhibitory activity of strain YM8 against additional species of phytopathogenic fungi

The strong inhibitory activity against *AF* mycelia and conidia prompted us to further test whether YM8 could inhibit the growth of other devastating pathogenic fungi that infect plants. Eight additional phytopathogenic fungal species, including *A. parasiticus, A. niger, A. alternate, B. cinerea, F. graminearum, F. oxysporum, M. fructicola*, and *S. sclerotiorum*, each of which can infect a wide range of plant families, were assayed with YM8 in sealed Petri dishes. Photos of the assay plates were taken 5 dpi. As shown in Figure 7B, none of the strain YM8-treated fungi grew, indicating complete inhibition. These results demonstrate that strain YM8 has a wide spectrum of growth inhibition activity against phytopathogenic fungi from different genera.

## Discussion

*Aspergillus* fungi produce carcinogenic aflatoxins. Therefore, the control of these microbes is considered to be fundamentally important in global food production and storage systems. Here, we characterized the YM8 strain of the marine bacterial species *S. algae* for use as a bio-control agent to control the growth of aflatoxigenic *Aspergillus* pathogens. Biological assays and chemical profiling of bioactive compounds revealed that a set of VOCs emitted by the YM8 strain were responsible for strong inhibition of *Aspergillus* growth and aflatoxin production. This inhibition activity was effective in both *in vitro* and in plant (maize, peanut) assays. Our results demonstrate that the volatiles produced by this microorganism can control the growth of *Aspergillus* spp. and thereby reduce the associated aflatoxin contamination of crops and stored food/feed.

DMTS was the most highly abundant volatile compound emitted by strain YM8 and was one of two most active compounds; it completely inhibited *AF* mycelial growth and conidial germination. This compound has recently been identified as an antifungal compound from plants (*Brassica oleracea*) (Valette et al., 2003) and other microorganisms such as *Streptomyces* sp. *Serratia* sp., and *Pseudomonas* sp. against different pathogens (*Rhizoctonia solani, Agrobacterium tumefaciens*, and *Penicillium italicum*) (Kai et al., 2007; Dandurishvili et al., 2011; Boukaew et al., 2013) indicating that this compound may have a generically active antifungal activity toward a common target shared by both pathogenic bacterium and fungi. This compound was produced in relatively low amounts by the microorganisms reported to date, whereas the *Shewanella* strain YM8 examined in this study produces a large quantity of DMTS; the high abundance of the DMTS may make a major contribution to its antifungal activity.

The remaining five VOCs selected for individual antifungal assays each had unique structural features and significantly inhibited the *AF* growth. 2,4-bis (1,1-dimethylethyl)-phenol was the most active compound assayed (Figure 4). It was recently identified as an antimicrobial compound from *Streptomyces* spp. (Elleuch et al., 2012; Saravana Kumer et al., 2014) and from plants (Rangel-Sánchez et al., 2014). Butylated hydroxytoluene has previously been extracted from plants and approved by the FDA of the United States as a commercial fungicide (Torres et al., 2014). Nonane was previously identified as a plant-derived antimicrobial compound (Saroglou et al., 2007). Two compounds, 2,4-dimethyl-oxazole and 2-dodecanol, have not been previously reported to possess antimicrobial activity. It has been suggested that hydroxyl groups of aromatic compounds and fatty acids can enter the fungal membrane and orient into the aqueous phase by hydrogen bonding and by alignment of non-polar carbon chain to the lipid phase by dispersion forces (Voda et al., 2004; Kim et al., 2007). The dispersion forces cause the disturbance of the fluidity of the cell membranes and thus disrupt the growth of fungi (Kubo et al., 1995; Voda et al., 2004; Kim et al., 2007). It is conceivable that a set of these compounds emitted from a single organism may generate synergistic antifungal activity. Strain YM8, which is capable of simultaneously synthesizing at least 15 VOC compounds, displays outstanding inhibition activity against *AF* and all of the other phytopathogenic fungi assayed. Further inhibition kinetics studies of different combinations of VOCs against aflatoxigenic *Aspergillus* fungi and other agronomically-important pathogens will provide new information on efficient use of this strain and mechanisms underlying the inhibition.

SEM analyses revealed the ultra-structural patterns of the key infection structures of *AF* on peanuts after treatment with VOCs emitted from strain YM8. *Aspergillus* spp. are air-borne fungi. During *AF* colonization of plant materials, conidia germinate and grow into mycelia whose apical cells subsequently develop into conidiophores; numerous conidia are then produced at the tips of conidiophores and these are dispersed through the air to initiate new infection cycles. VOCs from YM8 severely damaged conidia and completely inhibited conidial germination and thus abolished mycelial growth and conidiophore development. These VOCs can radically prevent fungal colonization and can eliminate aflatoxin production. The SEM images of our study provide further visual evidence for the effectiveness of the strain YM8 in controlling aflatoxins in food/feed samples. LC-ESI-MS analysis of aflatoxin content showed the inhibition activity of strain YM8 against aflatoxin production in agricultural samples. The AFB1 content of untreated control peanut and maize samples was higher than the maximum limit set by the WHO (5 μg/kg for AFB1) and the European Union (4 and 2 μg/kg for total AFs and AFB1, respectively) (Passone and Etcheverry, 2014). When treated with YM8, aflatoxin production was completely inhibited and undetectable through the methods used. This was the case for all of the different water activity levels tested.

Marine sediments cover more than 70% of the Earth's surface. Deep subsurface sediments have been estimated to contain more than 10^30^ bacterial and archaeal cells-over half of the biomass on Earth (Colin, 2011). Such environments are the exclusive habitat of many species of microorganisms, and many of these species can generate biologically active compounds. Over the past decade, marine microbes have been acknowledged as a significant and untapped source for novel bioactive compounds (Javed et al., 2011). Novel and structurally distinct secondary metabolites have been isolated from marine microorganisms and used as drugs in clinical trials (Javed et al., 2011). The marine *Shewanella* strain YM8 produces not only known antimicrobial agents but also bioactive compounds that have not been reported to date. Further screening and investigation will undoubtedly uncover more microorganisms from sea sediments that produce novel bioactive compounds.

Bacterium-emitted VOCs have advantages for the control of air-borne pathogens such as *Aspergillus* pathogens when compared to other bio-control strategies that require direct physical contact with pathogens. The advantages of VOCs over direct contact methods are particularly pronounced in the storage of food/feed products, as such storage often occurs in environments with controlled atmospheres. VOCs can quickly evaporate into the air and inhibit the growth of pathogens within a given volume of air. Further, there may be no or fewer volatile compound residues left on treated food/feed samples that would enter into food/feed chains as compared to other bio-agents that directly contact samples. Thus, the use of VOCs for controlling toxigenic pathogens infecting food/feed commodities could be considered as a potentially safe and environment-friendly strategy. Compared to extraction from plants, the extraction of VOCs from microorganisms has advantages that include easy large scale culturing in fermentation facilities under optimized conditions, shorter production times, and greater cost-effectiveness. Obviously, strain YM8 will need to be further studied for optimization of culture conditions and volatile production to preferentially produce the most active VOCs for specific uses.

The ability to completely inhibit the growth of nine different fungal species by strain YM8 suggests that the VOCs produced by this marine bacterium act against a shared target in these fungi. The nine fungal species are from different genera and have diverse genetic compositions, life cycles, and basic structures. *F. graminearum* and *A. alternate* also produce various mycotoxins when they infect agricultural samples in the field and during storage. The common interaction pattern between YM8 and different pathogenic fungi may serve as an ideal system for the further dissection of the molecular mechanisms of fungal growth inhibition and for the development of broad spectrum bio-control agents for agricultural applications. In summary, we identified a marine bacterium *Shewanella* strain YM8 that produces a set of VOCs with inhibition activity against mycelial growth and conidial germination of aflatoxigenic *Aspergillus* pathogens. The bioactive VOCs produced by strain YM8 include the well-known antimicrobial compounds DMTS and 2,4-bis(1,1-dimethylethyl)-phenol, the registered fungicide butylated hydroxytoluene, and several novel bioactive compounds. Assays with maize and peanut samples demonstrated that VOCs emitted from YM8 completely inhibited the growth of *Aspergillus* fungi and prevented aflatoxin biosynthesis. SEM images revealed severely damaged conidia and no formation of conidiophores or mycelia on *AF* inoculated samples. These novel findings demonstrate the high sensitivity of *Aspergillus* and eight other species of fungi to marine bacterial volatiles and indicates new strategies for the effective control of these pathogens and associated mycotoxins in both production systems and during storage. We envision that strain YM8 may serve as the foundation for both basic and applied research efforts in agriculture and biotechnology in the future.

### Conflict of interest statement

The authors declare that the research was conducted in the absence of any commercial or financial relationships that could be construed as a potential conflict of interest.
