# Supplementary material for: The Shewanella algae strain YM8 produces volatiles with strong inhibition activity against Aspergillus pathogens and aflatoxins
Source: Front Microbiol. 2015 Oct 6;6:1091. doi: 10.3389/fmicb.2015.01091 (PMC4594021; doi:10.3389/fmicb.2015.01091)
Supplement: Supplementary file 1 [file Table1.DOC]

**Supplementary information**

**The *Shewanella algae* strain YM8 produces volatiles with strong inhibition activity against *Aspergillus* pathogens and aflatoxins**

An-Dong Gong^1,2^, He-Ping Li^1,3^, Jing-Bo Zhang^1,2^, Ai-Bo Wu^4^, Wei-Jie He^1,3^, Qing-Song Yuan^1,2^, Jing-De He^1,2^, Yu-Cai Liao^1,2,5^*

^1^ Molecular Biotechnology Laboratory of Triticeae Crops, Huazhong Agricultural University, Wuhan 430070, People’s Republic of China, ^2^ College of Plant Science and Technology, Huazhong Agricultural University, Wuhan 430070, People’s Republic of China, ^3^ College of Life Science and Technology, Huazhong Agricultural University, Wuhan 430070, People’s Republic of China, ^4^ Key Laboratory of Food Safety Research Institute for Nutritional Sciences, Shanghai Institutes for Biological Sciences, Chinese Academy of Sciences, Shanghai 200031, People’s Republic of China, ^5^ National Center of Plant Gene Research (Wuhan), Huazhong Agricultural University, Wuhan 430070, People’s Republic of China.

**^*^ Correspondence should be addressed to:**

Dr. Yu-Cai Liao

College of Plant Science and Technology

Huazhong Agricultural University, Wuhan 430070

People’s Republic of China

Email: yucailiao@mail.hzau.edu.cn

**Table S1. Authentic reference standard** **compounds were purchased from companies and used in the *in vitro* assays against *Aspergillus flavus*.** All of these compounds were detected in the volatiles emitted from *Shewanella* strain YM8.

| ID | Compounds | Sources | CAS No. | Purity (%) |
| --- | --- | --- | --- | --- |
| 1 | Dimethyl trisulfide | Sigma-Aldrich | 03658-80-8 | ≥98.0 |
| 3 | 2,4-dimethyl-oxazole | Tokyo chemical industry | 07208-05-1 | ≥98.0 |
| 4 | Butylated Hydroxytoluene | J&K chemical industry | 00128-37-0 | ≥98.5 |
| 8 | 2-Dodecanol | Sigma-Aldrich | 06175-49-1 | ≥99.0 |
| 9 | Nonane | J&K chemical industry | 00111-84-2 | ≥99.0 |
| 10 | 2,4-bis(1,1-dimethylethyl)-phenol | J&K chemical industry | 00096-76-4 | ≥99.5 |
